# In vitro properties of designed antimicrobial peptides that exhibit potent antipneumococcal activity and produces synergism in combination with penicillin

**DOI:** 10.1038/srep09761

**Published:** 2015-05-18

**Authors:** Cheng-Foh Le, Mohd Yasim Mohd Yusof, Hamimah Hassan, Shamala Devi Sekaran

**Affiliations:** 1 Department of Medical Microbiology, Faculty of Medicine, University of Malaya, Kuala Lumpur, Malaysia; 2 School of Pharmacy, Faculty of Science, University of Nottingham Malaysia Campus, Semenyih, Selangor, Malaysia

## Abstract

Antimicrobial peptides (AMPs) represent a promising class of novel antimicrobial agents owing to their potent antimicrobial activity. In this study, two lead peptides from unrelated classes of AMPs were systematically hybridized into a series of five hybrid peptides (DM1- DM5) with conserved N- and C-termini. This approach allows sequence bridging of two highly dissimilar AMPs and enables sequence-activity relationship be detailed down to single amino acid level. Presence of specific amino acids and physicochemical properties were used to describe the antipneumococcal activity of these hybrids. Results obtained suggested that cell wall and/or membrane targeting could be the principal mechanism exerted by the hybrids leading to microbial cell killing. Moreover, the pneumocidal rate was greater than penicillin (PEN). Combination treatment with both DMs and PEN produced synergism. The hybrids were also broad spectrum against multiple common clinical bacteria. Sequence analysis showed that presence of specific residues has a major role in affecting the antimicrobial and cell toxicity of the hybrids than physicochemical properties. Future studies should continue to investigate the mechanisms of actions, *in vivo* therapeutic potential, and improve rational peptide design based on the current strategy.

Antimicrobial peptides (AMPs) have attracted much attention as the most promising next-generation antibiotics owing to its rapid and broad spectrum antimicrobial activity, Generally, they are short polycationic peptides of low molecular weight (usually made up of 12 to 50 amino acid residues), containing 50% hydrophobic amino acid residues, amphipathic, and can be found naturally in both animals and plants^1^,^2^. These small and rapid acting peptides exhibit broad spectrum antimicrobial activity against a diverse array of gram-positive and gram-negative bacterial species, fungi, as well as certain protozoan parasites and enveloped viruses^3^. They are able to function without memory or high specificity^4^.

Although wide sequential and structural variances of AMPs have been documented, the antimicrobial potential of AMPs is frequently recognized by a set of common traits which define the universal physicochemical properties of active peptides^2^. Presence of multiple cationic charges and high hydrophobicity represent the principal factors for AMPs to be active as antimicrobials^5^,^6^,^7^,^8^,^9^. The cationicity of AMPs represents the primary factor governing the initial contact with the oppositely charged bacterial surface via electrostatic interactions to facilitate subsequent actions^10^,^11^,^12^. Furthermore, the presence of specific amino acids at the precise position in the peptide chain is equally crucial for the expression of antimicrobial activity. This probably happens as the amino acids, each with its unique functional group, possess a great variety of physicochemical properties. Additionally, allocation of these amino acids at precise position in the peptide chain would ensure the structural integrity and stability of AMPs required for specific interaction with the targets be conserved. Due to the limitation in the number of possible sequence combinations, this also implies that there are limitations in the maximal antimicrobial activity achievable for any given peptide with definite chain length. For these reasons, systematic alteration of specific amino acids to modify the physicochemical properties of AMPs represent a straightforward yet effective approach towards understanding the influence of these factors on the antimicrobial activity of AMPs. Nevertheless, such considerations should be AMPs family-specific as it is unrealistic to expect two entirely unrelated AMPs families with high sequence diversity to exhibit similar antimicrobial spectrum based solely on the physicochemical values without considering the unique sequence identity that defines the specific antimicrobial activity of the AMPs.

Various strategies in designing novel synthetic analogues of AMPs have been described. One widely used method is sequence-based approach which correlates with antimicrobial activity in relation to the presence of specific amino acid/fragment at specific position. This approach is more direct and simple in design and does not usually require high-level computational technique. Ueno *et al*. designed a group of cationic AMPs based on a conservative strategy via acid-amide substitution (i.e. Glu to Gln, Asp to Asn)^13^. These substitutions will not cause major conformational alteration as compared to the parent peptide and thus helps preserve the integrity of the original structure to the highest level^13^. Interestingly, the newly generated peptides NP1P, NP2P, and NP3P showed marked increased in antibacterial activity against the gram-positive *Staphylococcus aureus*, *Bacillus subtilis*, and *Micrococcus luteus* as well as the gram-negative *Pseudomonas aeruginosa, Salmonella typhimurium*, *Escherichiae coli*, and *Serratia marcescens*^13^. On the other hand, Pasupuleti *et al*. reported that end-tagging AMPs with hydrophobic amino acids Trp or Phe enhanced antimicrobial activity against both *S. aureus* and *E. coli*. The high potency of the peptide was suggested to be due to high bacterial binding which caused bacterial cell lysis^13^. Besides, several of the synthetic AMPs designed by Chou *et al*. based on four physicochemical parameters including charge, hydrophobicity, hydrophobic moment, and polar angle exhibited high selectivity against *Vibrio* spp.^14^.

The current study aims at designing novel synthetic AMPs utilizing the peptide fragments hybridization strategy. The gradually truncated fragments from two unrelated AMP templates were designed into a series of five 13 amino acids in length hybrids. In this way, the analogous peptides under this hybrid series will be closely similar and the sequence-activity relationship can thus be investigated systematically. Moreover, an important aspect of drug-drug interaction and synergism was determined in combination with the beta-lactam antibiotic penicillin to understand the potential beneficial effect over standalone treatment.

## Results

### DM3 showed potent antipneumococcal and broad spectrum antibacterial activity

Peptide sequences and physicochemical properties of the hybrids are listed under Table 1. The DMs displayed strong antipneumococcal activity against all (100%) sixty pneumococcal isolates tested irrespective of the PEN susceptibility of the isolates (Table 2). Among the three PEN-susceptibility groups, the minimum inhibitory concentration (MIC) ranges differed by no more than two-fold dilution for the respective peptides. DM3 was the most potent antipneumococcal peptide among the hybrids, marking the lowest MIC levels against the penicillin-resistant *Streptococcus pneumoniae* (PRSP), penicillin-intermediate *S. pneumoniae* (PISP), and penicillin-susceptible *S. pneumoniae* (PSSP). The overall MIC range for DM3 was 7.81–62.5 μg/ml. As compared to DM3, the overall MICs were two-fold higher for both DM4 and DM5 (15.63–125 μg/ml) and four-fold higher for DM1 (31.3–250 μg/ml). For DM1, DM3, DM4, and DM5, the lowest and highest detectable MICs against *S. pneumoniae* were generally differed by four- to eight-fold except for DM2 which encompassed a larger range (7.81–250 μg/ml, 32-fold).

The hybrids exhibited potent antibacterial activity against multiple gram-positive and gram-negative bacteria. Notably, DM3 showed high antibacterial activity in the range of 7.81–62.5 μg/ml against the seven bacteria tested including *Klebsiella pneumoniae* (DM3 MIC = 62.5 μg/ml) (Table 3). *K. pneumoniae* was the least susceptible strain to the DMs among all bacteria tested. The antibacterial MICs of DM4 and DM5 ranged from 31.3–62.5 μg/ml, however, higher MIC levels were reported against *Enterococcus cloaceae*, *K. pneumoniae*, and *S. aureus*. As compared, the MIC of DM1 was equal to or two-fold higher than DM4 and DM5 against the respective strains. DM3 appeared to have higher gram-positive selectivity over gram-negative bacteria by as high as sixteen-fold difference in MIC (methicillin-resistant *S. aureus*, MRSA – 7.81 μg/ml; *P. aeruginosa* – 125 μg/ml).

### DMs showed greater pneumocidal kinetics than PEN

**Figure 1:**
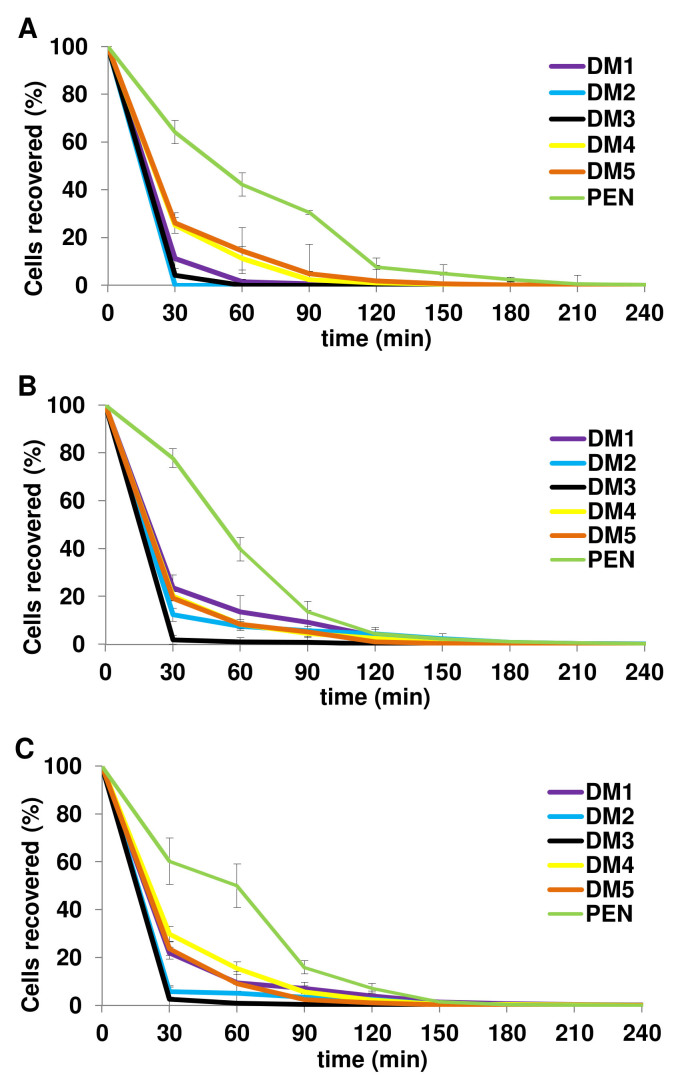
Percentage (%) of pneumococcal CFU recovered following treatment with peptides and PEN. At the specific time intervals, each (A) PRSP, (B) PISP, and (C) PSSP at 5 × 10^6^ CFU/ml was treated with 2× MIC of the respective peptides/PEN. Viable cells posttreatment were enumerated using plate count method. The pneumocidal rates of the five DMs were 48.5%, 61.3%, 64.6%, 42.4%, and 44.4% higher than PEN at 30 min posttreatment, respectively.

Substantial reductions in viable pneumococci treated with the five DMs were recorded (Figure 1). Immediately at the first time point post treatment (t = 30 min), the percentages of DMs-treated pneumococci recovered were less than 30% irrespective of PEN susceptibility of the isolates. DM2 and DM3 displayed the highest pneumocidal rates among the hybrids at which the proportions of colony forming unit (CFU) recovered at 30 min post treatment were <6.0% for all three groups of pneumococci (except 12.4% by DM2-treated PISP). Notably, DM2-treated PRSP was completely killed within the first 30 min post treatment. This strongly indicates that although the pneumocidal activity varied among the respective DMs, large proportions of the pneumococcal cells were rapidly cleared within the first 30 min and the CFU recovered continue to drop until eventual elimination. This is in sharp contrast to PEN-treated pneumococcal cells which remained at 60–78% viability examined at the same time point. The differences in killing kinetics against PRSP, PISP, and PSSP over PEN were 52.9%, 54.2%, and 38.4% respectively at an average of 48.5% for DM1; 64.1%, 65.4%, and 54.4% respectively at an average of 61.3% for DM2; 60.0%, 76.0%, and 57.7% respectively at an average of 64.6% for DM3; 39.0%, 57.8%, and 30.5% respectively at an average of 42.4% for DM4; 38.1%, 58.5%, and 36.5% respectively at an average of 44.4% for DM5. The results clearly demonstrated that the pneumocidal activities of DMs were dramatically greater than the conventional antibiotic PEN.

### DMs induced extensive cellular damaged leading to pneumococcal cell death

**Figure 2:**
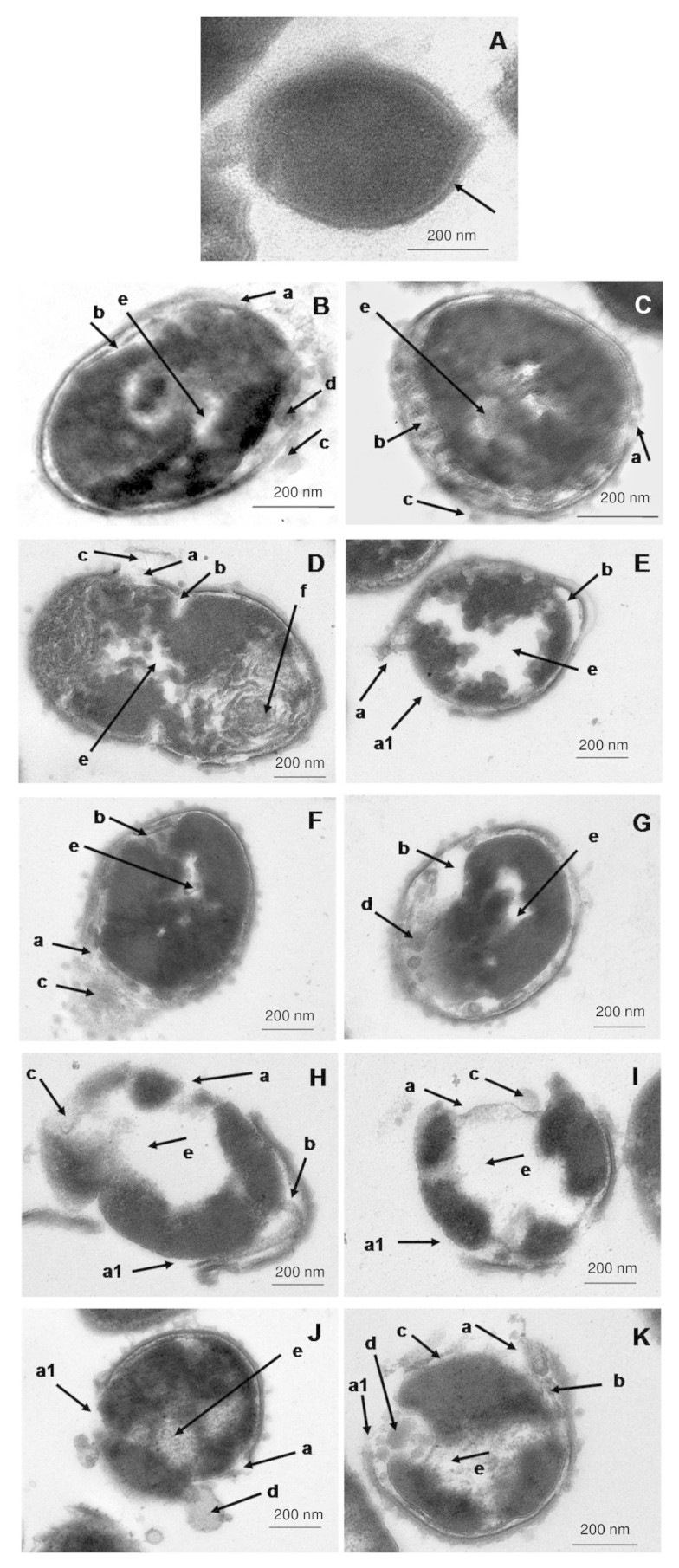
TEM images of PRSP treated with DM1 – DM5. (A) Untreated control showing the normal coccoidal shape of *S. pneumoniae* while severe cell morphological changes following treatment with (B & C) DM1, (D & E) DM2, (F & G) DM3, (H & I) DM4, and (J & K) DM5-treated pneumococcal cells were evident: (arrow a) cell wall/membrane breakages, (arrow a1) loss of cell wall, (arrow b) large cavity between cell wall and plasma membrane, (arrow c) leakage of intracellular contents, (arrow d) breakdown of cytoplasm into inclusion bodies, (arrow e) loss of cytoplasm, and (arrow f) filamentation of intracellular contents. Bar indicates 200 nm.

The changes in pneumococcal cell morphology induced by the five DMs on *S. pneumoniae* were imaged using Transmission electron microscopy (TEM). As shown in Figure 2, the untreated cell has an intact cell wall and cytoplasmic membrane that maintains the coccoidal shape of pneumococcus. The cytoplasm was densely packed and capsular polysaccharide was visible as a thin layer covering the outer layer of the cell (black arrow, Figure 2A). Following treatment with each DMs, severe cellular damages and extensive morphological changes of the pneumococcal cells were evident. In particular, cell wall and/or cytoplasmic membrane breakages were observed (arrow a). Loss of cell wall fragments were noted especially with DM4- and DM5-treated pneumococci. This exposed the cytoplasmic membrane and caused the “balding” appearance of the cells (arrow a1). Detachment of cell wall has led to the formation of large cavity space between the cell wall and cytoplasmic membrane (arrow b). With the loss of structural support from cell wall and the weakened cytoplasmic membrane, the cells became distorted, enlarged, and irregular in shape. Leakage of intracellular contents has then ensued (arrow c). The release of cytoplasmic contents seems to have occurred through breakdown and subsequent formation of cytoplasmic-containing inclusion bodies as in DM1, DM3, and DM5-treated cells (arrow d, Figure 2B, G, K). Bulging of cytoplasmic membrane leading to formation of inclusion bodies has also been noted in DM5-treated cells (arrow d, Figure 2J). Interestingly, the intracellular contents of DM2-treated pneumococci appeared filamentous and stacked at one pole of the cell (arrow f, Figure 2D). Additionally, large halos observed in the cytoplasm of all these treated cells could probably be due to breakdown/loss of cytoplasmic contents (arrow e, Figure 2B–K) leading to cell death and eventually appeared as an empty “shell”.

### DMs – PEN combinations produced synergism

The *in vitro* synergism effects of peptide-peptide and peptide-PEN combinations were tested using the checkerboard dilution method. Interestingly, combinations of PEN with all five DMs displayed synergism at fractional inhibitory index (FIC) ≤ 0.5 against all three groups of *S. pneumoniae* irrespective of PEN susceptibility of the isolates (Table 4). Among the peptide-peptide combinations, synergism was noted between DM5 and DM1, DM2, and DM4.

### DMs produced varying degrees of hemolytic and cytotoxicity

DM1, DM4, and DM5 displayed no hemolytic activity at the range of concentrations tested (HC_10_ > 250 μg/ml, H_max_ < 1.0%) (Table 5). DM1 was also the least cytotoxic peptide among the hybrids, which showed IC_50_ of >220 μg/ml against NL20 cell line and ≥195.0 μg/ml against A549 cell line (Table 6). For DM4 and DM5, the overall cell cytotoxicity was lower against NL20 cell line than A549 cell line. For DM4, the IC_50_ of NL20 cells at 24 hrs, 48 hrs, and 72 hrs were 66.9 μg/ml, 64.3 μg/ml, and 97.5 μg/ml higher than A549 cells. Similarly, DM5-treated NL20 cells were 82.2 μg/ml, 81.9 μg/ml, and 90.0 μg/ml higher than DM5-treated A549 cells. However, no major difference in cell viability was observed at the I_max_ level (except for DM4-treated cells at 72 hrs). DM3 (HC_10_ = 52.7 μg/ml) was the most hemolytic and cytotoxic peptide among the hybrids. However, the hemolytic level was considered moderate as at the highest concentration of 250 μg/ml tested, DM3 caused only 39% hemolysis. Besides, the cytotoxicity of DM3 against NL20 cell line was found to decrease over prolonged duration from IC_50_ of 68.5 ± 11.0 μg/ml at 24 hrs to 91.5 ± 7.6 μg/ml and 112.2 ± 8.8 μg/ml at 48 hrs and 72 hrs posttreatment, respectively. Similarly, the cytotoxicity against A549 cells was reduced from IC_50_ of 56.0 ± 9.3 μg/ml at 24 hrs to 80.0 ± 1.7 μg/ml and 96.2 ± 4.4 μg/ml at 48 hrs and 72 hrs posttreatment, respectively. DM2 which was the second most hemolytic hybrid peptide (HC_10_ = 114.3 μg/ml) showed only minimal level of cytotoxicity against NL20 cell line while the cell viability of A549 cell line was found to be decreasing over extended treatment duration from IC_50_ of >250 μg/ml at 24 hrs to 197.7 μg/ml and 165.0 μg/ml at 48 hrs and 72 hrs posttreatment, respectively.

## Discussion

Synthetic derivatives of AMPs designed using various approaches have been increasingly documented in recent years. Intense efforts have been grounded in developing novel synthetic AMPs with improved antimicrobial activity as the new antibiotic candidates. In the present study, we set out a new strategy to systematically hybridize peptide fragments from two distinctive classes of AMPs characterized by source organisms, AMPs families, sequence similarity, and sequence identity. From our extensive database and literature searches, CP10A and A4 which matched to our selection criteria were chosen. Both of these templates were equal in length (13 amino acids) to eliminate the peptide length effect. In this way, the antimicrobial activity can be specifically explained in relation to the fragment, sequence, and physicochemical properties of AMPs. These peptides were originated from two different hosts (CP10A, indolicidin, *Bos Taurus*; A4, *E. coli*, also resembles Aurein 1.2 from *Litoria aurea*) and the sequence similarity between these two templates was negligible (2/13 residues, 15.4%). In addition, we are particularly interested in CP10A among other short peptides owing to its high percentage of Trp residues (38.46%). Conversely, this sequence identity was not found in peptide A4 (0% Trp). With such high level of dissimilarity, one would expect hybridizing these peptides would unavoidably disrupt the integrity of either templates rendering the generation of inactive hybrids. Remarkably, the designed DMs exhibited strong antipneumococcal activity and were broad spectrum. Based on the results obtained, we have proposed the potential mechanisms induced by the DMs leading to bacterial cell death.

The antimicrobial activity of the hybrids was assessed against *S. pneumoniae* as the model bacterium. The DMs showed potent antipneumococcal activity including the PISP and PRSP isolates. Pneumocidal kinetics was high and considerably greater than PEN. With such rapid bactericidal activity of DMs, this implies that the bacterial cells could have insufficient time to activate resistance mechanisms that are frequently metabolically exhaustive and time-consuming. Remarkably, we also showed that co-treatment of DMs with PEN was indeed superior than the individual agents against all three groups of PRSP, PISP, and PSSP. Apart from that, the DMs also displayed broad-spectrum antibacterial activity against multiple clinically important strains irrespective of the gram types of the bacteria. The closely comparable antipneumococcal and antibacterial MIC ranges of the hybrids suggests that the concentration/dose range to be used against pneumococci will likely be effective against the other bacterial species too. These hybrids were essentially non-hemolytic as seen with DM1, DM4, and DM5. Although DM3 was the most hemolytic peptide among the DMs, the level was moderate and equivalent to a maximum of 15% hemolysis only considering the effective MIC level against *S. pneumoniae*.

Systematic hybridization of template fragments provides an excellent way to describe the sequence-activity and physicochemical-activity relationships of peptides. For instances, DM2 can be considered as the K5I (K to I substitution at position 5) variant of DM1 while DM3 represents the A7K variant of DM2. Based on the overall MIC, the antipneumococcal potency of the hybrids can be ranked, from highest to lowest, as DM3 > DM2 > DM4 and DM5 > DM1. However, we found no association between net charge (NetC), charge density (ChD), and hydrophobic ratio (HR) to antipneumococcal activity of the hybrids. This is because DM3 and DM1, the most and least potent peptides, respectively have the same NetC of +5, ChD of 0.38, and HR of 53% which fell between the highly charged DM4 and DM5 and the lower charge DM2. The only physicochemical property difference is the hydrophobicity values (total hydrophobic value, THV and Grant Average of Hydropathy, GRAVY). The higher hydrophobicity value could have contributed to the enhanced antipneumococcal potency in DM3. Another explanation is the presence of Ile^5^ and K^7^W^8^. Single residual substitution of DM1 to K5I substantially improved the antipneumococcal activity of DM2 and further A7K substitution has generated DM3. Moreover, replacing W^8^ in DM3 to other amino acids dramatically reduced the antipneumococcal activity as observed in DM4 and DM5. Nevertheless, hydrophobicity is of least impact to DM4 and DM5 as the high charge value could have masked or reduced the influence of hydrophobicity rendering indifference in their antimicrobial activity and cell toxicity. This indicates that the effect of hydrophobicity is negligible at position 9 of the peptides. A previous study conducted by Tsai *et al*. had also determined that Lys^13^ and Arg^22^ residues were critical for the anticandidal activity of Histatin-5. Replacing the Lys^13^ and Arg^22^ residues with Glu^13^ and Gly^22^ residues caused a dramatic reduction in the anticandidal MICs of Histatin-5 variant m68^15^. Furthermore, Subbalakshmi *et al*. suggested that tryptophan residue was responsible for the hemolytic activity of indolicidin analogs but has no contribution to the antimicrobial activity^16^. Nevertheless, the hemolytic and cell cytotoxicity levels appeared to be directly proportionate to the antimicrobial level of the peptides. This suggests that the current amino acid alterations are insufficient to dissociate prokaryotic and eukaryotic cells selectivity.

Morphologically, extensive cellular changes observed with DMs-treated pneumococcal cells clearly demonstrated the detrimental effects induced by DMs at the supra-MIC levels. Although the detailed underlying mechanisms might differ between the respective peptides, for instances, DM1 caused direct leakage of intracellular contents while DM5 caused the bulging and eventual formation of cytoplasm-containing inclusion bodies through exocytosis, two major mechanisms involved were cell wall and cytoplasmic membrane disruptions. Interestingly, DM3 induced cytoplasmic disintegration leading to formation of small inclusion bodies while the cell wall remained intact. This suggests that DM3 could have gained entry to the cytoplasm by translocation through the membrane without affecting the cell wall.

Altogether, this leads to the hypothesis that the DMs are potential cell wall and/or plasma membrane targeting-peptides based on the findings that the antimicrobial activities of the DMs were (1) independent of PEN susceptibility and thus the molecular resistance mechanisms involved in PEN resistance, (2) independent of pneumococcal serotype and thus ruled out the specific capsular polysaccharide-targeting potential of the DMs, (3) high bactericidal rate immediately following treatment and thus may not be dependent upon the metabolic mechanisms of *S. pneumoniae* for its killing activity, (4) broad spectrum against a wide variety of bacteria of both gram types and thus the difference in cell wall composition has no significant effect on the selectivity of DMs, and (5) images from TEM provide clear evidence showing severe damages particularly on cell wall and plasma membrane of pneumococci. Therefore, cell wall/membrane disruption appears to be the primary antimicrobial mechanism involved. With the co-presence of PEN in synergism treatment, PEN would inhibit the PBPs and weakens the cell wall of *S. pneumoniae* thus facilitating the peptide molecules to act more rapidly onto the bacterial cell wall and other cellular components. This could have reduced the time and number of peptide monomers required per pneumococcal cell to initiate killing. However, we do not exclude the possibility that other cooperative mechanisms might have contributed to the antimicrobial activity of the DMs, too. This is because AMPs could be membrane-active when present at high concentration but turn to target intracellular organelles at low concentration, or both, as shown by Friedrich *et al.*^17^,^18^.

The systematic fragment hybridization strategy described in the present study would be particularly useful in sequence-base novel AMPs design. This technique enables a three-in-one designing strategy whereby the peptides can be analysed by looking at the fragment-activity, residue-activity, and/or physicochemical-activity relationships. The principal rule is the design should follow a systematic fashion. Moreover, this method allows bridging of two unrelated peptides creating hybrids resembling the partial identity of both templates. Although the underlying antimicrobial mechanisms of the hybrids may differ considerably between analogues even with only single amino acid modification, the bridging would potentially allow identification of fragments/stretch of amino acids crucial for the biological activity of AMPs. Hence, this strategy should form the basis and expanded to include candidates from other classes of AMPs in future rational design of hybrid peptides. The specific antimicrobial actions of our hybrids will need further investigations to allow better understanding of the mechanisms of actions involved. Future studies should continue to focus on the dynamics and complex peptide interactions leading from initial peptide-membrane contact to the cascading events such as translocation, intracellular interaction/disruption events, and others. Additional imaging modalities such as Atomic Force Microscopy and Confocal Microscopy would benefit the observation of the interactions. Furthermore, *in vivo* testing of the DMs will provide a better understanding of the therapeutic efficacy, synergism, and toxicity in animal model mimicking human bacterial infection.

In conclusion, AMPs represent a promising class of antimicrobial agents for the development of novel antimicrobial agents. It is important to highlight that each AMPs family is unique and designing peptide derivatives should be highly family-specific. Physicochemical properties and/or sequence crucial for a particular class of AMPs may or may not be applicable to other classes of AMPs. More supporting data is needed to improve the designs further. Of note, the beneficial outcome from combination treatment with AMPs and conventional antibiotics against bacterial infection is evident and should be explored.

## Methods

### Peptides design and synthesis

Five hybrid peptides (DM1, DM2, DM3, DM4, and DM5) were designed based on two previously reported AMPs-derived peptides: the peptide A4 (Protein Data Bank ID: 1VM4) which is the nontoxic bacterial membrane anchor/antibacterial peptide aurein 1.2^19^ and the indolicidin-derived peptide CP10A (Protein Data Bank ID: 1HR1)^17^ with sequence modifications. Val^6^ of Peptide A4 was substituted with Trp^6^ to generate A4-M (GLFDIWKKLVSDF) while Ala^3^,^10^ of 1HR1 were substituted with Trp^3^ and Arg^10^ to generate CP10A-M (ILWWKWAWWRWRR). Subsequently, the N-terminus of A4-M and C-terminus of CP10A-M variable length peptide fragments were aligned and hybridized in a systematic approach: DM1 was designed using the first four residues at the N-terminus of A4-M (GLFD) and hybridized with the 5^th^–13^th^ C-terminal residues fragment (KWAWWRWRR-NH_2_) of CP10A-M. DM2, DM3, DM4, and DM5 were designed using the same approach by hybridization of the first five/six (produced identical sequences as both A4-M and CP10A-M have Trp at position 6), seven, eight, and nine N-terminal residues of A4-M to the seven/eight, six, five, and four residues of CP10A-M C-terminal fragments. The four amino acids at the N- and C-termini were unaltered from the template sequences. Hence, the hybrids have C-terminal amidation as in CP10A. All peptides were synthesized using 9-fluorenylmethoxycarbonyl solid phase peptide synthesis chemistry to >90% purity (Genscript, USA) and validated using High Performance Liquid Chromatography and Mass Spectrometry.

### Peptides physicochemical analytical tools

Five physicochemical parameters describing the charge and hydrophobicity properties of the peptides were computed using the ExPASy ProtParam tools (http://web.expasy.org/protparam/) and Antimicrobial Peptide Database (http://aps.unmc.edu/AP/main.php). These parameters included NetC, ChD, HR, THV, and GRAVY. NetC was calculated by subtracting the sum of positively-charged amino acids (Arg, Lys, His) by the sum of negatively-charged amino acids (Asp, Glu). C-terminal amidation was assigned with one positive charge (+1). ChD was calculated by dividing NetC with the chain length of peptide. HR is the percentage of hydrophobic amino acid (Ile, Val, Leu, Phe, Cys, Met, Ala, Trp) in the peptide chain. THV was computed by summation of hydrophobicity value for each residue based on the Kyte and Doolittle hydropathy index^20^. GRAVY was the mean hydrophobicity value of the peptide, calculated by dividing THV by the total number of residues.

### Bacterial cultures and assay medium

All bacterial strains were obtained from Department of Medical Microbiology, University of Malaya. Clinical isolates were previously obtained from University of Malaya Medical Centre and maintained in the laboratory. Sheep blood agar was used for pneumococcal cultivation purpose. Nutrient agar was used for cultivating *E. coli* ATCC 25922, *S. aureus* ATCC 25923, and *P. aeruginosa* ATCC 15442, *Acinetobacter baumanii* ATCC 15308, and one clinical isolate for each MRSA, *E. cloacae, Citrobacter* spp., and *K. pneumoniae*. For antimicrobial testing, Mueller-Hinton agar (MHA) and cationically-adjusted MHB (CAMHB) were prepared according to the Clinical and Laboratory Standard Institute guidelines^21^. MHA for *S. pneumoniae* was supplemented with 5% (v/v) defibrinated sheep blood and 5% laked horse blood for CAMHB. All bacterial cultures were stored in multiple vials in brain heart infusion broth supplemented with 10% (v/v) glycerol at −80°C to avoid repeated freeze-thawed cycles on the cells. All freeze-stocked strains were passaged twice prior to experimentation.

### Broth microdilution assay

Procedures were performed to determine the MIC of peptides according to the CLSI guidelines^21^ against 60 pneumococcal isolates which consisted of 20 isolates each for PRSP, PISP, and PSSP. Strains were grown for 18–24 hrs at 37°C under 5% CO_2_. Direct suspension of the colonies were made in CAMHB and adjusted to OD_625_ 0.08–0.1 which corresponds to 1 ~ 2 × 10^8^ CFU/ml followed by serial ten-fold dilutions to give 1 × 10^6^ CFU/ml. Fifty microliters of bacterial suspension was then aliquoted to 96-well round bottom microtiter plates containing equal volume of serially diluted peptides to give final concentrations of peptides encompassing the range of 1.96–250 μg/ml. The MIC value for inactive peptides producing no inhibition in the range tested was denoted as >250 μg/ml. The plates were incubated for 18–24 hrs at 37°C under 5% CO_2_. MIC was read as the concentration of peptide producing complete inhibition on the visible growth of the test organism. Results were pooled from three independent experiments. MIC range was defined as the range of concentrations where the peptides produce detectable antimicrobial activity. Effective percentage (EP) was the proportion of pneumococcal isolates inhibited within the MIC range. Since the isolates tested were obtained prior to 2008 where major revision in antibiotic susceptibility breakpoints were introduced, thus the pneumococci were defined according to the PEN susceptibility breakpoint (prior 2008) which corresponds to the PEN (oral penicillin V) breakpoint of the revised criteria.

The broad spectrum antibacterial activity of the peptides against a panel of eight clinically important bacteria was also determined, this includes *S. aureus* ATCC 25923, *E. coli* ATCC 25922, *P. aeruginosa* ATCC 15442, *A. baumanii* ATCC 15308, and one clinical isolate each for MRSA, *E. cloacae, Citrobacter* spp., and *K. pneumoniae*.

### Bacterial killing assay

This assay was performed to evaluate the killing kinetics of peptides^22^. One PRSP, PISP, and PSSP isolate were each grown and prepared to 5 × 10^6^ CFU/ml. Each isolate in CAMHB was challenged with peptides at two times the respective MIC values. At the indicated time intervals (0, 30, 60, 120, 150, 180, 210, and 240 min; “0” indicate pretreatment), 10 μl of suspension was removed and immediately serially diluted in cold phosphate-buffered saline to arrest further reaction before spread-plated on MHA to obtain viable colony counts. The percentage cell recovered was calculated by dividing the surviving CFU of the treated cells over the surviving CFU of the untreated control cells at the respective time points. Results were pooled from three independent experiments and expressed as mean ± standard deviation (SD).

### Transmission electron microscopy

An overnight culture of PRSP on sheep blood agar was passaged twice and directly suspended in CAMHB. As sample for TEM required high cell density of about 10^8^ to 10^10^ to be viewable^23^, the bacteria was prepared to 5 × 10^10^ CFU/ml and treated with supra-concentration of peptides at 8 mg/ml for four hours at 37°C under 5% CO_2_. Cells treated with only water was served as the untreated control. For TEM sample preparation, standard protocol provided by the Electron Microscopic Unit, Faculty of Medicine, University of Malaya was followed. The cells were washed twice with CAMHB before overnight fixation in 4% (v/v) glutaraldehyde, two times postfix washes with cacodylate buffer, incubate, two hours incubation with osmium tetroxide buffer (OsO_4_ 1: 1 cacodylate), and washed twice with cacodylate buffer before overnight incubation in the same buffer. Next, the samples were washed twice with double distilled water, 10 min of uranyl acetate incubation, and washed twice with double distilled water before subjected to dehydration by gradual ethanol series: 35% for 10 min, 50% for 10 min, 70% for 10 min, 95% for 15 min, and three rounds of absolute (100%) ethanol for 15 min. Following this, samples were incubated with two rounds of propylene oxide for 15 min, propylene oxide 1:1 Epon for 1 hr, propylene oxide 1:3 Epon for 2 hrs, overnight incubation with Epon, embedded in Agar 100 resin at 37°C for five hours, and maintained in 60°C until viewing. Ultrathin sectioning were prepared on Reichert Ultramicrotome, copper grids 3.05 mm (300 square mesh) (Agar Scientific), and stained with ethanol-based uranyl acetate and lead citrate for 5 min. The prepared samples were viewed with Leo Libra 120 under standard operating conditions.

### Checkerboard dilution assay

Synergism assay was performed using the checkerboard method^24^ with minor modification. In order to prepare a range of concentrations which allows simultaneous detection of antagonism, indifference/additive, and synergism, the assay was performed in such a way that each column contained a fixed 0.25× MIC of the first peptide and 12 serial two-fold dilutions of the second peptide at each row beginning at 8× MIC. This yielded 12 peptide-peptide (peptide A: peptide B) combinations at varying ratios from 1:128 to 16:1. The combinational effect of peptides was defined according to the FIC index, whereby
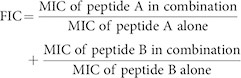


Two peptides were antagonistic if FIC > 4.0, indifference if 0.5 < FIC ≤ 4.0, and synergism if ≤0.5^24^. Each peptide and PEN pair was tested against one isolate for each PRSP, PISP, and PSSP in different combinations. Bacteria were prepared according to broth microdilution assay without laked horse blood to allow better visual observation of well clearance.

### Hemolytic activity

Hemolytic assay was performed as described^25^. Freshly drawn human erythrocytes were rinsed three times with PBS and resuspended in PBS to 4% (v/v). One hundred microliters of the suspension was added to 96-well microtiter plate containing equal volume of peptides to give final concentrations of peptides encompassing the range of 1.96–250 μg/ml. PBS and 0.1% (v/v) Triton-X 100 were used as 0% and 100% hemolytic control respectively. Plates were incubated at 37°C for 1 hr. Subsequently, the plate was centrifuged and the supernatant was transferred to a new plate. The release of hemoglobin in the supernatant was monitored at absorbance of 450 nm using Glomax Multidetection system (Promega, USA). Results were pooled from three independent experiments and expressed as mean ± SD. HC_10_ and HC_50_ were defined as the peptide concentrations causing 10% and 50% hemolysis on human erythrocytes, respectively. H_max_ was the percentage hemolysis observed at the maximum concentration as defined throughout this study (all peptides 250 μg/ml, PEN 4 μg/ml).

### Cytotoxicity against human cell lines

Both the NL20 human lung bronchial normal epithelial cell line and the A549 human lung alveolar adenocarcinoma epithelial cell line were used for cell cytotoxicity tests. Besides investigating the cytotoxicity of peptides, differences in cell viability between the two cell lines can be used to evaluate the cell line selectivity and to assess potential anticancer potential of the peptides. NL20 cell line was grown in Ham's F12 medium and A549 cell line was grown in Roswell Park Memorial Institute medium. Both media were supplemented with fetal bovine serum to 10% (v/v) as growth medium or 2% (v/v) as maintenance medium in cell cytotoxicity testing. The assay was performed as described by Lee *et al*.^26^. NL20 cells was seeded overnight at 3 × 10^4^ cells/well in 96-well cell culture-treated flat bottom microtiter plate and treated with serial dilutions of peptide at a final concentration of 1.95–250 μg/ml. PEN was tested from 0.03–4 μg/ml, the same range used to classify the susceptibility of pneumococcal isolates in this study. Test using A549 cells followed the same procedures with 1 × 10^4^ cells/well. Plates were incubated at 37°C under 5% CO_2_ for 24, 48, and 72 hrs. Cell viability was detected by using CellTiter 96® AQueous Non-Radioactive Cell Proliferation assay (Promega, USA) and the colorimetric changes were read with Glomax multidetection system (Promega, USA) at OD 490 nm. Results were pooled from three independent experiments and expressed as mean ± SD. IC_50_ was defined as the peptide concentration which resulted in 50% cell viability. I_max_ was the percentage of cell viable treated with peptides/PEN at the maximum concentration as defined throughout this study (all peptides 250 μg/ml, PEN 4 μg/ml).

## Author Contributions

C.F.L., M.Y.M.Y., H.H. and S.D.S. designed the research and performed experiments; C.F.L. and S.D.S. analyzed data; C.F.L., M.Y.M.Y., H.H. and S.D.S. wrote the paper.

## Additional Information

**How to cite this article**:Le, C.-F., Yusof, M.Y.M., Hassan, H.& Sekaran, S.D. In vitro properties of designed antimicrobial peptides that exhibit potent antipneumococcal activity and produces synergism in combination with penicillin. *Sci. Rep.* **5**, 9761; doi: 10.1038/srep09761 (2015).

## Figures and Tables

**Table 1:** Physicochemical properties of DMs

**Table 2:** MICs of DMs against S. pneumoniae of varying PEN-susceptibility

Peptide	PRSP			PISP			PSSP			Overall MIC (μg/ml)	Overall EP (%)
	MIC (μg/ml)	no. of isolates (EP %)		MIC (μg/ml)	no. of isolates (EP %)		MIC (μg/ml)	no. of isolates (EP %)			
DM1	31.3–125	20	(100.0)	31.3–125	20	(100.0)	62.5–250	20	(100.0)	31.3–250	100.0
DM2	15.63–250	20	(100.0)	7.81–250	20	(100.0)	15.63–250	20	(100.0)	7.81–250	100.0
DM3	15.63–62.5	20	(100.0)	7.81–62.5	20	(100.0)	7.81–62.5	20	(100.0)	7.81–62.5	100.0
DM4	31.3–125	20	(100.0)	15.63–125	20	(100.0)	15.63–125	20	(100.0)	15.63–125	100.0
DM5	31.3–125	20	(100.0)	15.63–125	20	(100.0)	15.63–125	20	(100.0)	15.63–125	100.0

**Table 3:** Broad spectrum antibacterial activity of DMs against eight common human bacterial pathogens

Peptide	MIC (μg/ml)							
	Gram-positive		Gram-negative					
	*S. aureus* ATCC 25923	MRSA	*E. coli* ATCC 25922	*P. aeruginosa* ATCC 15442	*A. baumanii* ATCC 15308	*E. cloaceae*	*Citrobacter* spp.	*K. pneumoniae*
DM1	125	62.5	62.5	125	62.5	125	31.3	>250
DM2	31.3	62.5	250	>250	31.3	>250	62.5	>250
DM3	15.63	7.81	62.5	125	15.63	31.3	15.63	62.5
DM4	62.5	62.5	62.5	31.3	31.3	250	31.3	>250
DM5	125	62.5	31.3	31.3	31.3	125	31.3	250

**Table 4:** FIC index of peptide-peptide/penicllin combinations against PRSP, PISP, and PSSP

Combination		PRSP		PISP		PSSP	
		FIC index	Interpretation^a^	FIC index	Interpretation^a^	FIC index	Interpretation^a^
DM1	DM2	0.75	Additive/indifference	0.75	Additive/indifference	0.63	Additive/indifference
	DM3	0.75	Additive/indifference	0.75	Additive/indifference	0.63	Additive/indifference
	DM4	0.75	Additive/indifference	0.63	Additive/indifference	0.63	Additive/indifference
	DM5	**0.50**	**Synergy**	0.63	Additive/indifference	0.63	Additive/indifference
	PEN	**0.38**	**Synergy**	**0.50**	**Synergy**	**0.16**	**Synergy**
DM2	DM3	0.75	Additive/indifference	0.75	Additive/indifference	1.13	Additive/indifference
	DM4	0.75	Additive/indifference	0.63	Additive/indifference	1.13	Additive/indifference
	DM5	**0.50**	**Synergy**	0.63	Additive/indifference	0.63	Additive/indifference
	PEN	**0.38**	**Synergy**	**0.50**	**Synergy**	**0.25**	**Synergy**
DM3	DM4	0.75	Additive/indifference	0.63	Additive/indifference	1.13	Additive/indifference
	DM5	0.75	Additive/indifference	0.75	Additive/indifference	1.13	Additive/indifference
	PEN	**0.38**	**Synergy**	**0.50**	**Synergy**	**0.19**	**Synergy**
DM4	DM5	**0.50**	**Synergy**	0.75	Additive/indifference	0.63	Additive/indifference
	PEN	**0.28**	**Synergy**	**0.50**	**Synergy**	**0.28**	**Synergy**
DM5	PEN	**0.25**	**Synergy**	**0.50**	**Synergy**	**0.19**	**Synergy**

^a^FIC ≤ 0.05 denotes synergism, >0.5–4 denotes indifference, and >4 denotes antagonism.

Synergistic pairs are in bold face.

FIC, fractional inhibitory concentration.

**Table 5:** Hemolytic activity (Percent ± SD) of DMs on human erythrocytes

Peptide	Hemolytic activity (μg/ml) Percent hemolysis ± SD		
	HC_10_	HC_50_	H_max_ (%)
DM1	>250	>250	0.5 ± 0.1
DM2	114.3 ± 26.1	>250	13.9 ± 2.0
DM3	52.7 ± 5.5	>250	39.0 ± 2.5
DM4	>250	>250	0.6 ± 0.1
DM5	>250	>250	0.9 ± 0.4
PEN	>4	>4	0.6 ± 0.3

HC_10_ and HC_50_ were defined as the concentrations of peptide causing 10% and 50% hemolysis on human erythrocytes, respectively.

H_max_ was defined as the percentage (%) hemolysis of peptide at the highest concentration tested (all peptides - 250 μg/ml; PEN - 4 μg/ml).

**Table 6:** Cell cytotoxicity of DMs and the templates on NL20 and A549 cell lines

Peptide	Percent cytotoxicity ± SD											
	NL20 cell line (μg/ml)						A549 cell line (μg/ml)					
	24 hrs		48 hrs		72 hrs		24 hrs		48 hrs		72 hrs	
	IC_50_	I_max_	IC_50_	I_max_	IC_50_	I_max_	IC_50_	I_max_	IC_50_	I_max_	IC_50_	I_max_
DM1	221.8 ± 7.8	35.3 ± 6.6	235.7 ± 12.9	42.2 ± 8.6	242.2 ± 7.3	45.9 ± 4.3	215.3 ± 19.7	38.6 ± 6.2	195.0 ± 25.2	39.8 ± 7.7	>250	66.2 ± 7.2
DM2	>250	64.3 ± 7.0	>250	53.3 ± 9.1	241.5 ± 9.0	46.8 ± 4.0	>250	50.7 ± 7.7	197.7 ± 12.6	24.0 ± 6.3	165 ± 14.2	12.0 ± 4.1
DM3	68.5 ± 11.0	13.3 ± 2.1	91.5 ± 7.6	24.0 ± 7.6	112.2 ± 8.8	12.4 ± 4.5	56.0 ± 9.3	4.2 ± 3.2	80.0 ± 1.7	4.4 ± 2.4	96.2 ± 4.4	3.7 ± 2.1
DM4	160.2 ± 11.8	6.9 ± 6.8	167.3 ± 5.9	5.9 ± 2.5	205.0 ± 7.0	30.2 ± 2.9	93.3 ± 5.1	8.5 ± 3.8	103.0 ± 17.5	8.9 ± 1.4	107.5 ± 16.3	2,9 ± 2.7
DM5	179.2 ± 3.8	7.3 ± 2.4	188.2 ± 4.9	8.3 ± 4.2	191.7 ± 3.5	9.7 ± 5.4	97.0 ± 9.8	9.0 ± 1.0	106.3 ± 15.7	7.8 ± 3.8	101.7 ± 9.5	8.0 ± 3.2
PEN	>4	105.2 ± 0.2	>4	106.1 ± 6.2	>4	88.3 ± 0.4	>4	95.0 ± 0.4	>4	97.3 ± 0.4	>4	91.1 ± 6.0

IC_50_ was defined as the concentration of peptide which reduced cell viability to 50%.

I_max_ was defined as the percentage (%) cell viability tested at the highest concentration of peptides (all peptides - 250 μg/ml; PEN - 4 μg/ml).
